# Supplementary material for: Beneficial dose-dependent effects of Ag nanoparticles on germination do not compromise growth and metabolic profiles of Capsicum annuum seedlings
Source: PeerJ. 2025 Sep 9;13:e19974. doi: 10.7717/peerj.19974 (PMC12428529; doi:10.7717/peerj.19974)
Supplement: Supplemental Information 1 [file peerj-13-19974-s001.docx]

**Table S1.** Survival test for germination rates of *Capsicum annuum* domesticated (DM) and wildtype (WT) varieties after 14 days of treatment. Chi-square test.

| **Plant type** | **Test** | | **Chi square** | ***d.f.*** | ***P*** |
| --- | --- | --- | --- | --- | --- |
| Domesticated | Log-Rank | 11.35 | | 3 | **0.01** |
|  | Wilcoxon | 20.22 | | 3 | **0.0002** |
| Wildtype | Log-Rank | 2.65 | | 3 | 0.449 |
|  | Wilcoxon | 1.02 | | 3 | 0.7975 |
