# Supplementary material for: Beneficial dose-dependent effects of Ag nanoparticles on germination do not compromise growth and metabolic profiles of Capsicum annuum seedlings
Source: PeerJ. 2025 Sep 9;13:e19974. doi: 10.7717/peerj.19974 (PMC12428529; doi:10.7717/peerj.19974)
Supplement: Supplemental Information 2 [file peerj-13-19974-s002.docx]

**Table S2.** Estimates of ANOVA of morphological traits of 28-days after germination plants, as a function of *Capsicum annuum* variety (wild vs cultivated), treatment of silver nanoparticles exposure, and its interaction.

| **Trait** | **Source** | ***d.f*.** | **ss** | **F ratio** | ***P*** |
| --- | --- | --- | --- | --- | --- |
| Shoot length (cm) | Plant type | 1 | 3.42 | 6.57 | **0.02** |
|  | Treatment (Ag ppm) | 3 | 2.90 | 1.86 | 0.16 |
|  | Plant type × Treatment (Ag ppm) | 3 | 4.30 | 2.76 | 0.06 |
| Root length (cm) | Plant type | 1 | 34.04 | 2.47 | 0.13 |
|  | Treatment (Ag ppm) | 3 | 33.85 | 0.82 | 0.49 |
|  | Plant type × Treatment (Ag ppm) | 3 | 48.73 | 1.18 | 0.33 |
| Total length (cm) | Plant type | 1 | 59.05 | 4.06 | **0.05** |
|  | Treatment (Ag ppm) | 3 | 51.67 | 1.18 | 0.33 |
|  | Plant type × Treatment (Ag ppm) | 3 | 81.51 | 1.87 | 0.16 |
| Shoot wet mass (g) | Plant type | 1 | 0.03 | 25.52 | **<0.0001** |
|  | Treatment (Ag ppm) | 3 | 0.00 | 0.65 | 0.59 |
|  | Plant type × Treatment (Ag ppm) | 3 | 0.00 | 1.13 | 0.35 |
| Root wet mass (g) | Plant type | 1 | 0.03 | 5.63 | **0.02** |
|  | Treatment (Ag ppm) | 3 | 0.03 | 2.32 | 0.09 |
|  | Plant type × Treatment (Ag ppm) | 3 | 0.01 | 0.75 | 0.53 |
| Total wet mass (g) | Plant type | 1 | 0.11 | 11.78 | **<0.0001** |
|  | Treatment (Ag ppm) | 3 | 0.03 | 1.07 | 0.37 |
|  | Plant type × Treatment (Ag ppm) | 3 | 0.01 | 0.37 | 0.77 |
| Shoot dry mass (g) | Plant type | 1 | 0.00 | 11.44 | **<0.0001** |
|  | Treatment (Ag ppm) | 3 | 0.00 | 0.86 | 0.47 |
|  | Plant type × Treatment (Ag ppm) | 3 | 0.00 | 2.19 | 0.11 |
| Root dry mass (g) | Plant type | 1 | 0.00 | 2.16 | 0.15 |
|  | Treatment (Ag ppm) | 3 | 0.00 | 0.73 | 0.54 |
|  | Plant type × Treatment (Ag ppm) | 3 | 0.00 | 0.64 | 0.59 |
| Total dry mass (g) | Plant type | 1 | 0.00 | 4.66 | **0.04** |
|  | Treatment (Ag ppm) | 3 | 0.00 | 0.80 | 0.50 |
|  | Plant type × Treatment (Ag ppm) | 3 | 0.00 | 0.81 | 0.50 |
