# Supplementary material for: Beneficial dose-dependent effects of Ag nanoparticles on germination do not compromise growth and metabolic profiles of Capsicum annuum seedlings
Source: PeerJ. 2025 Sep 9;13:e19974. doi: 10.7717/peerj.19974 (PMC12428529; doi:10.7717/peerj.19974)
Supplement: Supplemental Information 3 [file peerj-13-19974-s003.docx]

**Table S3.** Estimates of ANOVA of morphological traits of 42 days after germination plants, as a function of *Capsicum annuum* variety (wild vs cultivated), treatment of silver nanoparticles exposure, and its interaction.

| **Trait** | **Source** | ***d.f.*** | | | | **ss** | **F ratio** | ***P*** |
| --- | --- | --- | --- | --- | --- | --- | --- | --- |
| Root length (cm) | Plant type | | 1 | | 23.26 | | 3.67 | 0.06 |
|  | Treatment (Ag ppm) | | | 3 | 4.75 | | 0.25 | 0.86 |
|  | Plant type × Treatment (Ag ppm) | | | 3 | 48.12 | | 2.53 | 0.07 |
| Shoot length (cm) | Plant type | | | 1 | 50.40 | | 202.21 | **<0.0001** |
|  | Treatment (Ag ppm) | | | 3 | 0.60 | | 0.80 | 0.50 |
|  | Plant type × Treatment (Ag ppm) | | | 3 | 0.93 | | 1.25 | 0.31 |
| Total length (cm) | Plant type | | | 1 | 142.13 | | 19.99 | **<0.0001** |
|  | Treatment (Ag ppm) | | | 3 | 4.39 | | 0.21 | 0.89 |
|  | Plant type × Treatment (Ag ppm) | | | 3 | 62.37 | | 2.92 | **0.05** |
| Shoot wet mass (g) | Plant type | | | 1 | 0.10 | | 8.72 | **<0.0001** |
|  | Treatment (Ag ppm) | | | 3 | 0.06 | | 1.79 | 0.17 |
|  | Plant type × Treatment (Ag ppm) | | | 3 | 0.04 | | 1.24 | 0.31 |
| Root wet mass (g) | Plant type | | | 1 | 0.02 | | 1.80 | 0.19 |
|  | Treatment (Ag ppm) | | | 3 | 0.12 | | 4.22 | **0.01** |
|  | Plant type × Treatment (Ag ppm) | | | 3 | 0.04 | | 1.30 | 0.29 |
| Total wet mass (g) | Plant type | | | 1 | 0.20 | | 5.30 | **0.03** |
|  | Treatment (Ag ppm) | | | 3 | 0.36 | | 3.16 | **0.04** |
|  | Plant type × Treatment (Ag ppm) | | | 3 | 0.15 | | 1.32 | 0.29 |
| Shoot dry mass (g) | Plant type | | | 1 | 0.00 | | 3.24 | 0.08 |
|  | Treatment (Ag ppm) | | | 3 | 0.00 | | 2.37 | 0.09 |
|  | Plant type × Treatment (Ag ppm) | | | 3 | 0.00 | | 1.06 | 0.38 |
| Root dry mass (g) | Plant type | | | 1 | 0.00 | | 4.13 | **0.05** |
|  | Treatment (Ag ppm) | | | 3 | 0.00 | | 2.17 | 0.11 |
|  | Plant type × Treatment (Ag ppm) | | | 3 | 0.00 | | 0.80 | 0.50 |
| Total dry mass (g) | Plant type | | | 1 | 0.00 | | 3.62 | 0.07 |
|  | Treatment (Ag ppm) | | | 3 | 0.00 | | 2.37 | 0.09 |
|  | Plant type × Treatment (Ag ppm) | | | 3 | 0.00 | | 1.02 | 0.40 |
