# Supplementary material for: Beneficial dose-dependent effects of Ag nanoparticles on germination do not compromise growth and metabolic profiles of Capsicum annuum seedlings
Source: PeerJ. 2025 Sep 9;13:e19974. doi: 10.7717/peerj.19974 (PMC12428529; doi:10.7717/peerj.19974)
Supplement: Supplemental Information 4 [file peerj-13-19974-s004.docx]

**Table S4.** ANOVA of total polyphenolic content on leaves of 28- and 42-days after germination plants, as a function of *C. annuum* variety (wild vs cultivated), treatment of silver nanoparticles exposure, and its interaction.

| **Trait** | **Source** | ***d.f.*** | **ss** | **F ratio** | ***P*** |
| --- | --- | --- | --- | --- | --- |
| TPC (mg/g) 28-days | Plant type | 1 | 0.69 | 1.10 | 0.30 |
|  | Treatment (Ag ppm) | 3 | 1.59 | 0.85 | 0.48 |
|  | Plant type × Treatment (Ag ppm) | 3 | 1.21 | 0.64 | 0.59 |
| TPC (mg/g) 42-days | Plant type | 1 | 0.00 | 0.00 | 0.96 |
|  | Treatment (Ag ppm) | 3 | 2.91 | 0.98 | 0.42 |
|  | Plant type × Treatment (Ag ppm) | 3 | 2.19 | 0.73 | 0.54 |
