# Supplementary material for: Beneficial dose-dependent effects of Ag nanoparticles on germination do not compromise growth and metabolic profiles of Capsicum annuum seedlings
Source: PeerJ. 2025 Sep 9;13:e19974. doi: 10.7717/peerj.19974 (PMC12428529; doi:10.7717/peerj.19974)
Supplement: Supplemental Information 5 [file peerj-13-19974-s005.docx]

**Table S5.** ANOVA of Chlorophyll content on leaves measured after 28 and 42 days after germination, as a function of *Capsicum annuum* variety (wild vs cultivated), treatment of silver nanoparticles exposure, and its interaction.

| **Trait** | **Source** | ***d.f.*** | **ss** | **F ratio** | ***P*** |
| --- | --- | --- | --- | --- | --- |
| ICC 28-days | Plant type | 1 | 113.63 | 6.20 | **0.02** |
|  | Treatment (Ag ppm) | 3 | 26.79 | 0.49 | 0.69 |
|  | Plant type × Treatment (Ag ppm) | 3 | 50.48 | 0.92 | 0.45 |
| ICC 42-days | Plant type | 1 | 1250.42 | 84.47 | **<.0001** |
|  | Treatment (Ag ppm) | 3 | 85.56 | 1.93 | 0.15 |
|  | Plant type × Treatment (Ag ppm) | 3 | 246.49 | 5.55 | **0.005** |
