# Supplementary material for: Beneficial dose-dependent effects of Ag nanoparticles on germination do not compromise growth and metabolic profiles of Capsicum annuum seedlings
Source: PeerJ. 2025 Sep 9;13:e19974. doi: 10.7717/peerj.19974 (PMC12428529; doi:10.7717/peerj.19974)
Supplement: Supplemental Information 6 [file peerj-13-19974-s006.docx]

**Table S6.** ANOVA results of root and shoot length of *C. annuum* at the start of exposure to AgNPs at 24, 48 and 72 hours.

| **Trait** | **Source** | **Estimate** | **Std error** | **t ratio** | ***p*** |
| --- | --- | --- | --- | --- | --- |
| Root 0 h | Plant type | 3.59 | 0.92 | 3.91 | **0.0012** |
|  | Treatment (Ag ppm) | -0.62 | 0.92 | -0.67 | 0.5103 |
|  | Plant type × Treatment (Ag ppm) | 0.73 | 0.92 | 0.8 | 0.4377 |
| Root 24h | Plant type | 3.83 | 0.92 | 4.16 | **0.0007** |
|  | Treatment (Ag ppm) | -0.72 | 0.92 | -0.78 | 0.445 |
|  | Plant type × Treatment (Ag ppm) | 0.59 | 0.92 | 0.64 | 0.5314 |
| Root 48h | Plant type | 4.02 | 0.93 | 4.31 | **0.0005** |
|  | Treatment (Ag ppm) | -0.88 | 0.93 | -0.94 | 0.3616 |
|  | Plant type × Treatment (Ag ppm) | 0.47 | 0.93 | 0.5 | 0.6218 |
| Root 72h | Plant type | 4.23 | 0.92 | 4.58 | **0.0003** |
|  | Treatment (Ag ppm) | -0.91 | 0.92 | -0.98 | 0.3416 |
|  | Plant type × Treatment (Ag ppm) | 0.50 | 0.92 | 0.55 | 0.5923 |
| Shoot 0 h | Plant type | 1.55 | 0.10 | 15.49 | **<.0001** |
|  | Treatment (Ag ppm) | 0.15 | 0.10 | 1.45 | 0.1656 |
|  | Plant type × Treatment (Ag ppm) | 0.19 | 0.10 | 1.87 | 0.0805 |
| Shoot 24h | Plant type | 1.65 | 0.10 | 16.1 | **<.0001** |
|  | Treatment (Ag ppm) | 0.12 | 0.10 | 1.21 | 0.2457 |
|  | Plant type × Treatment (Ag ppm) | 0.18 | 0.10 | 1.77 | 0.0964 |
| Shoot 48h | Plant type | 1.65 | 0.09 | 19.2 | **<.0001** |
|  | Treatment (Ag ppm) | 0.04 | 0.09 | 0.41 | 0.687 |
|  | Plant type × Treatment (Ag ppm) | 0.21 | 0.09 | 2.5 | **0.0238** |
| Shoot 72h | Plant type | 1.72 | 0.09 | 18.94 | **<.0001** |
|  | Treatment (Ag ppm) | 0.10 | 0.09 | 1.09 | 0.2933 |
|  | Plant type × Treatment (Ag ppm) | 0.25 | 0.09 | 2.79 | **0.0131** |
