# Supplementary material for: Beneficial dose-dependent effects of Ag nanoparticles on germination do not compromise growth and metabolic profiles of Capsicum annuum seedlings
Source: PeerJ. 2025 Sep 9;13:e19974. doi: 10.7717/peerj.19974 (PMC12428529; doi:10.7717/peerj.19974)
Supplement: Supplemental Information 7 [file peerj-13-19974-s007.docx]

**Table S7.** LS Means Differences Tukey HSD of the interaction between the plant type and treatment of the shoot length of the plants after 48 and 72 hours of exposure to AgNPs treatment.

| **Trait** | **Level** | **Least Sq Mean** | **Std error** |
| --- | --- | --- | --- |
| Shoot 48 h | Serrano,AgNPs | 8.1394 | 0.17 |
|  | Serrano,dH2O | 7.64 | 0.17 |
|  | SPCs,AgNPs | 4.4126 | 0.17 |
|  | SPCs,dH2O | 4.771 | 0.17 |
| Shoot 72h | Serrano,AgNPs | 8.5414 | 0.18 |
|  | Serrano,dH2O | 7.8368 | 0.18 |
|  | SPCs,AgNPs | 4.5926 | 0.18 |
|  | SPCs,dH2O | 4.9022 | 0.18 |
