# Supplementary material for: Beneficial dose-dependent effects of Ag nanoparticles on germination do not compromise growth and metabolic profiles of Capsicum annuum seedlings
Source: PeerJ. 2025 Sep 9;13:e19974. doi: 10.7717/peerj.19974 (PMC12428529; doi:10.7717/peerj.19974)
Supplement: Supplemental Information 8 [file peerj-13-19974-s008.docx]

**Table S8.** ANOVA results of the number of cells found in a certain phase of mitosis in roots of wild and domesticated *C. annuum* plants after 72 hours of exposure to a solution of silver nanoparticles (AgNP).

| **Trait** | **Source** | **Estimate** | **Std error** | **t ratio** | ***p*** |
| --- | --- | --- | --- | --- | --- |
| Prophase | Plant type | 0.00 | 0.00 | 2.34 | **0.032** |
|  | Treatment (Ag ppm) | 0.00 | 0.00 | -0.39 | 0.705 |
|  | Plant type × Treatment (Ag ppm) | 0.00 | 0.00 | -1.59 | 0.132 |
| Metaphase | Plant type | 0.00 | 0.00 | 1.74 | 0.101 |
|  | Treatment (Ag ppm) | 0.00 | 0.00 | -0.74 | 0.470 |
|  | Plant type × Treatment (Ag ppm) | 0.00 | 0.00 | -1.87 | 0.080 |
| Anaphase | Plant type | 0.00 | 0.00 | -0.71 | 0.489 |
|  | Treatment (Ag ppm) | 0.00 | 0.00 | -0.61 | 0.553 |
|  | Plant type × Treatment (Ag ppm) | 0.00 | 0.00 | 0.84 | 0.414 |
| Telophase | Plant type | 0.00 | 0.00 | 0.63 | 0.535 |
|  | Treatment (Ag ppm) | 0.00 | 0.00 | -1.39 | 0.185 |
|  | Plant type × Treatment (Ag ppm) | 0.00 | 0.00 | -1.32 | 0.206 |
| Interphase | Plant type | 0.00 | 0.00 | -1.17 | 0.261 |
|  | Treatment (Ag ppm) | 0.00 | 0.00 | 1.06 | 0.306 |
|  | Plant type × Treatment (Ag ppm) | 0.00 | 0.00 | 1.19 | 0.253 |
