# Supplementary material for: Beneficial dose-dependent effects of Ag nanoparticles on germination do not compromise growth and metabolic profiles of Capsicum annuum seedlings
Source: PeerJ. 2025 Sep 9;13:e19974. doi: 10.7717/peerj.19974 (PMC12428529; doi:10.7717/peerj.19974)
Supplement: Supplemental Information 9 — Each data point represents the average total wet mass, root wet mass and total length measured in plants subjected to different treatments with silver nanoparticles. The total length indicates both shoot and root length. [file peerj-13-19974-s009.pdf]

Figure S1. Total wet mass (g), Root wet mass (g) and Total length of domesticated and wild *C. annuum* plants after 42 days of germination at different AgNP concentrations (50, 100 and 250 ppm).

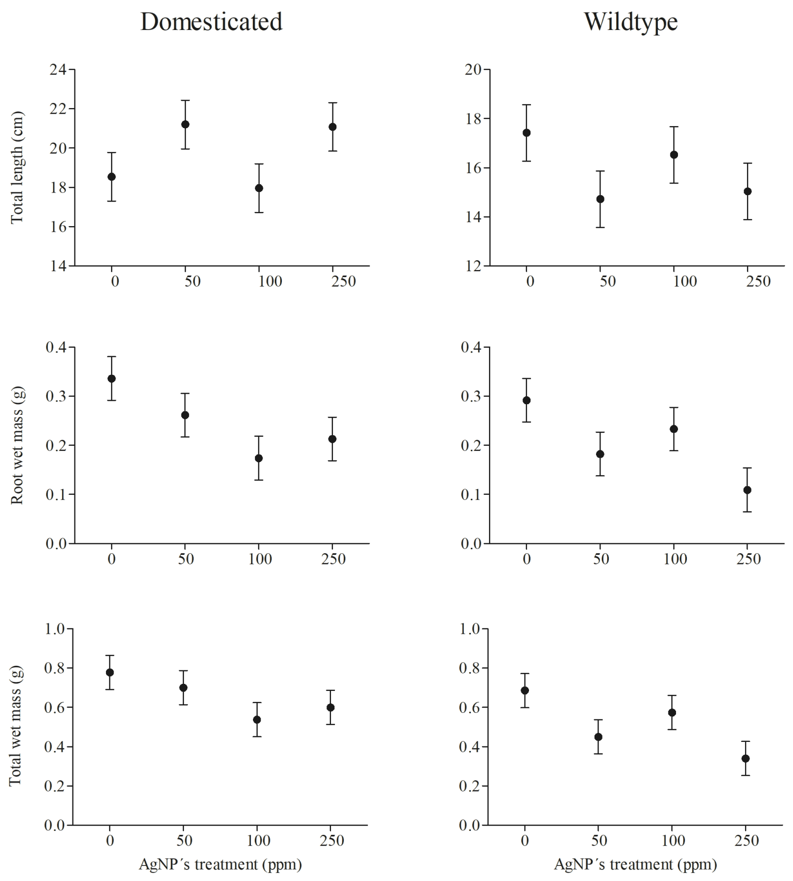

Commented [MOU1]: Borrar?
