# Supplementary material for: Beneficial dose-dependent effects of Ag nanoparticles on germination do not compromise growth and metabolic profiles of Capsicum annuum seedlings
Source: PeerJ. 2025 Sep 9;13:e19974. doi: 10.7717/peerj.19974 (PMC12428529; doi:10.7717/peerj.19974)

Figure S2. Total polyphenol content (TPC) of 28- and 42-days post-germination wild and domesticated plants with each silver nanoparticle (AgNPs) treatment (50, 100 and 250 ppm).

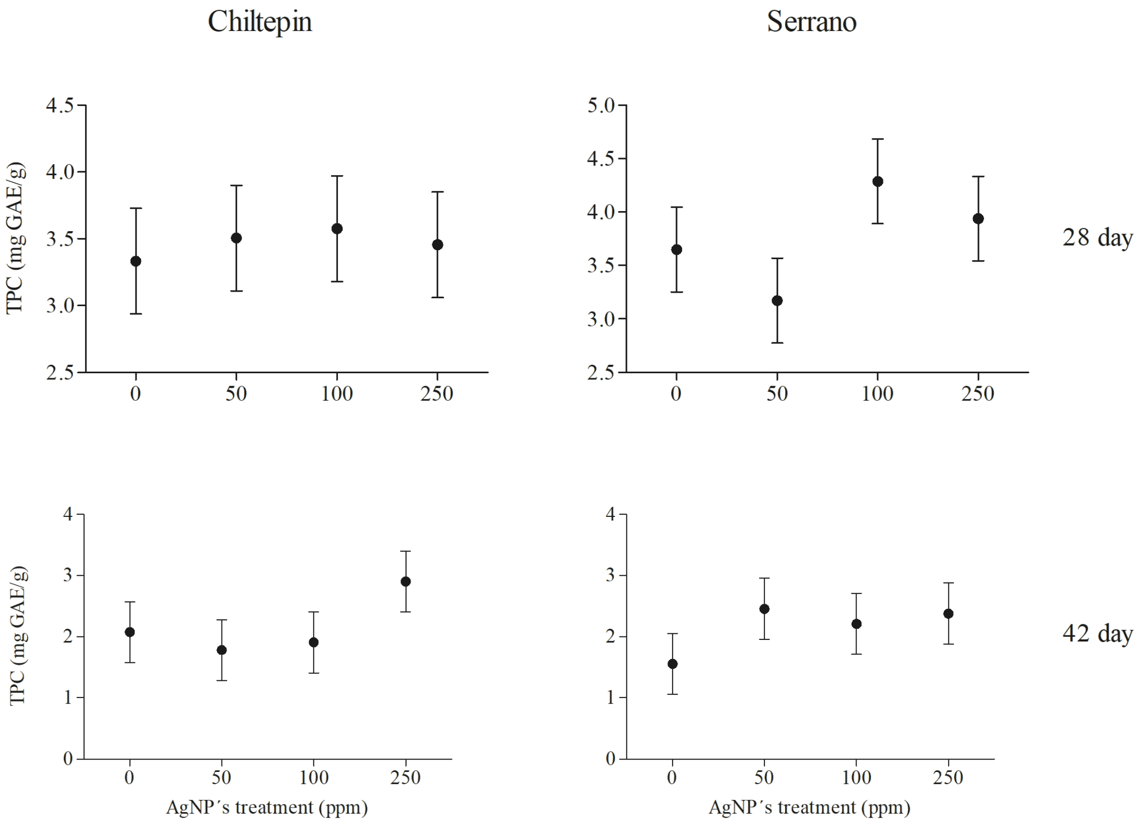

Supplement: Supplemental Information 10 — Each data point represents the average Total polyphenol content measured in plants subjected to different treatments with silver nanoparticles. The measurements were taken 28 and 42 days after germination. [file peerj-13-19974-s010.pdf]
